# Causes of Error in Reports Relative to the Physical Evidence of Assault on Young Girls

**Published:** 1884-06

**Authors:** 


### Causes of Error in Reports Relative to the Physical Evidence of Assault on Young Girls.

Prof. Brouardel (*Annales de Gynecologie*), says in referring to this matter: "A young girl may be troubled with vulvitis accompanied by a more or less abundant discharge. Her mother, naturally troubled about it, will question the girl closely and as it often happens will really, by her questions, suggest a kind of story which the imagination of the girl seizes upon and finally believes to be her own, though it may really have no foundation in fact. The incidents are connected perhaps with some boy or man who has been more or less in the company of the child, and thus there sometimes arises a false accusation which may have serious consequences. Hence the exact physiology of the female genital organs in their first conditions, together with such modifications in their general aspects as are most frequently found, should be thoroughly known to the practitioner if he would avoid very important errors. Three of the principal forms of hymen are susceptible of false interpretations. First is the circular hymen, in whose surrounding parts there exist in many cases certain folds which, upon a too rapid or cursory examination, might easily be taken for ridges, or cicatrices caused by previous ruptures. In holding the membrane tense, however, it will be observed that in cases where the membrane has remained entire, the tissue is continuous in appearance. The same hymens will often, owing to their elasticity and feeble resistive power, permit the entrance of the penis without rupturing at all. Then there is the hymen in form of a cross, and having two of its branches, at their extremities, attached to perfectly normal notches—so to speak—one at each side of the vagina. Care should be used not to mistake these bases of attachment for accidental ruptures. For the third form I will say that in certain cases the hymen, instead of constituting a continuous membrane perforated by two holes, presents the aspect of having originally been so constituted, while its appearance at the time of examination is that of two orifices incompletely separated by a loose fluctuating (*flottante*) membrane. This aspect is also not unlikely to create a false impression that there has been a rupture. The examination of the hymen may be rendered very difficult by the development of fatty tissue in the labiæ majoræ. In such cases the vulva becomes more deeply situated, and it is only in the centre of a sort of vulvular canal that the hymeneal membrane is found. One important fact should always be present in the mind of the expert. I refer to the possible cicatrization of the wound produced by the rupture of the hymen. There is in these cases only a sort of momentary defloration; an attentive examination will reveal a cicatrice upon the membrane in question and enable the expert to avoid an error in diagnosis. The vulva may be the seat of inflammations of a diverse nature, and also of ulcerations, two accidental conditions which may be developed together or separately. Inflammation, or vulvitis, may be found in three types, which may be described as strumous, traumatic, and blennorrhagic. Between these three imflammatory characteristics a clear differential diagnosis is difficult to establish. The physician can only pronounce upon them after several examinations. Strumous vulvitis takes on chronic appearances, but is capable of presenting acute exacerbations. Traumatic vulvitis, whatever may be the source of injury, declares itself three or four days after the action of the cause; its duration averages about fifteen days. In blennorrhagic vulvitis there is a period of incubation of about eight days. The inflammation is intense, accompanied by a burning heat of the vulva, and finally, the urethra is invaded, which is not the case—or very exceptionally so—in other forms of vulvitis. The lymphatic ganglia and vulvo-vaginal glands furnish but weak elements for diagnosis; their tumefication is proportioned to the intensity of the vulvitis, but gives little evidence as to its nature. The varieties of ulceration which may develop upon the vulva are many, some of a simple, and others of a venereal nature. Hard chancres, mucous patches and soft chancres belong to the latter category. Herpes, ulcerations due to a simple aphthous vulvitis, corrosive or diphtheritic, to the former. The elements of diagnosis are multiple, but unfortunately are uncertain. The "knowing how to wait" (*savoir attendre*), upon which M. Fournier insists with such good reason, is here of the highest importance. How many physicians have caused a condemnation of the innocent by pronouncing their opinion too quickly. M. Brouardel reports, among others, two interesting cases where errors have been made in these cases. In the first it was a question of aphthous vulvitis, complicated with gangrene, which was mistaken for violence exercised upon the genital organs. The second was a diphtheritic vulvitis in complication with laryngo-tracheal diphtheria in which a similar etiology had been falsely pronounced."—*Bulletin Gen. de Therap.*

